# Preoperative risk factors associated with patient outcomes following arthroscopic elbow contracture release

**DOI:** 10.1016/j.jseint.2026.101621

**Published:** 2026-01-12

**Authors:** Jacquelyn J. Xu, Brian O. Molokwu, Bradley A. Lezak, Roban Shabbir-Hussain, Tanzeel Sultan, C. Lucas Myerson, Mandeep S. Virk, Young W. Kwon

**Affiliations:** Division of Shoulder and Elbow Surgery, Department of Orthopedic Surgery, NYU Grossman School of Medicine, NYU Langone Orthopedic Hospital, NYU Langone Health, New York, NY, USA

**Keywords:** Elbow contracture, Elbow stiffness, Arthroscopic osteocapsulectomy, Osteocapsular arthroplasty, Ulnar nerve decompression, Patient-reported outcomes, Elbow arthroscopy

## Abstract

**Background:**

Elbow contractures can occur due to several causes including age-related degenerative disease, inflammatory conditions, and post-traumatic injury. The resulting pain and reduced range of motion can significantly interfere with activities of daily living and impede patient functionality. One treatment option for elbow contractures is arthroscopic contracture release. The purpose of this study is to evaluate the preoperative risk factors associated with inferior clinical outcomes following arthroscopic elbow contracture release.

**Methods:**

This retrospective cohort study included patients ≥18 years old who underwent arthroscopic elbow contracture release at a single institution between January 2015 and November 2024. Patients were included if they had preoperative and postoperative Patient-Reported Outcome Measurement Information System (PROMIS) Upper Extremity Function (P-UE), Pain Interference (P-Interference), and Pain Intensity (P-Intensity) scores collected at ≥6 months follow-up. Postoperative PROMIS scores were stratified into tertiles to identify patients with inferior clinical outcomes, defined as the lowest tertile for function and highest tertile for pain-related measures. Multivariate logistic regression analyses were performed to evaluate the association between preoperative patient factors and inferior postoperative outcomes. *P* values were adjusted for multiple comparisons using the Bonferroni correction. Postoperative complications, including recurrent contracture, neurological symptoms, and reoperations, were also recorded.

**Results:**

A total of 65 patients (47 males and 18 females) with a mean age of 52.2 years (range: 19-77) were followed for a mean of 18 months postoperatively for in-person assessments. Mean flexion improved from 122° (range, 80°-145°) to 127° (90°-150°) (*P* = .02), extension from 20° (0°-50°) to 14° (−5° to 45°) (*P* < .001), and the flexion-extension arc from 103° (30°-140°) to 113° (45°-145°) (*P* < .001). Supination and pronation remained stable (79°-81° and 85°-85°, respectively). PROMIS scores were collected at a mean follow-up of 51.6 (range: 7-131) months. Function (*P* = .01) and pain intensity scores (*P* = .01) both improved significantly following surgery. Female sex (odds ratio: 10.7; *P* = .02) was associated with low postoperative P-UE scores.

**Conclusion:**

Arthroscopic elbow contracture release can improve function and range of motion; however, outcomes may vary based on preoperative patient characteristics. Female sex may be associated with lower postoperative PROMIS function scores.

Elbow contractures commonly occur after a traumatic incident or due to primary osteoarthritis but may also occur following inflammatory conditions.^5^^,^^13^^,^^19^ This results in pain and reduced range of motion.^9^^,^^23^ While modest elbow contractures can be well tolerated, significant contractures can decrease functional capacity and interfere with activities of daily living. The first-line treatment is nonoperative management consisting of physical therapy and/or serial splinting to increase range of motion.^12^ Often, however, patients will fail conservative management and require surgical intervention.^2^^,^^16^

Contracture release via open capsulectomy or osteocapsular arthroplasty is a well-established surgical intervention to address stiffness and improve function by excising fibrotic capsular tissue and osteophytes and removing loose bodies. While improvements in motion are generally modest, predictable improvements in pain and functional capacity have been reported in these patients.^8^^,^^18^^,^^20^ Arthroscopic technique is an alternative option to open capsular release and is preferred for many surgeons due to its minimally invasive nature resulting in shorter recovery times with lower rates of complications.^10^^,^^14^^,^^22^

Despite the established efficacy of arthroscopic contracture release for the treatment of elbow stiffness, variable patient outcomes are observed due to complications such as permanent or transient nerve injuries and recurrent stiffness occurring in up to 10% of cases.^17^^,^^11^ Additionally, there is a paucity of data on risk factors associated with inferior outcomes in patients undergoing this procedure.

The primary purpose of this study was to evaluate preoperative factors associated with inferior patient-reported outcomes in patients undergoing arthroscopic elbow contracture release (ECR). A secondary objective was to assess the incidence of delayed-onset ulnar neuritis or neuropathy in ECR patients with preoperative elbow flexion <100° who did not undergo prophylactic ulnar nerve decompression (PUND).

## Methods

### Study design

This study was approved for exemption by our institution's institutional review board. This was a retrospective cohort study of prospectively collected data at an academic tertiary care center between January 2015 and November 2024 using Current Procedural Terminology codes 24149 and 29838. A detailed retrospective chart review was performed to confirm if patients underwent open or arthroscopic ECR. Inclusion criteria were patients over the age of 18 years at the time of procedure, arthroscopic ECR, and completion of preoperative and postoperative Patient Reported Outcome Measurement Information System (PROMIS) Upper Extremity Computer Adaptive Testing v2.0 (P-UE), Pain Interference (P-Interference), and Pain Intensity (P-Intensity) surveys at a minimum of 6 months postoperatively.^7^^,^^28^^,^^31^ Patients were excluded if they underwent open surgery.

### Surgical technique and postoperative rehabilitation

All procedures were performed by one of two fellowship-trained shoulder and elbow surgeons. Patients were positioned in the lateral decubitus position with the operative arm supported for elbow arthroscopy. Standard anteromedial and anterolateral portals were used to access the anterior compartment, where synovectomy and complete anterior capsular release were performed. Posterior compartment access was obtained through posterolateral and posterior central portals, followed by synovectomy and posterior capsular release. When indicated, the radiocapitellar joint and radial gutter were addressed through a lateral portal for débridement. Intraoperative range of motion was assessed following capsular release. Postoperatively, patients initiated supervised passive and active range of motion to tolerance within the first postoperative days, including elbow flexion–extension and forearm rotation, with gentle overpressure into extension and frequent home exercises. Range of motion was progressively advanced over the first 2-3 weeks, followed by gradual strengthening and return to functional activities, with adjunctive ice and compression as needed.

### Data collection

Surgical intervention was considered for patients with persistent pain and functional limitation who failed nonoperative management, which typically included supervised physical therapy (for 3-6 months) with stretching and, when appropriate, dynamic or static splinting. Diagnosis was established through clinical evaluation by one of the attending shoulder and elbow surgeons (Y.W.K. and M.S.V.) and confirmed by radiographic imaging (radiographs or computed tomography) of the elbow. Patient charts were reviewed to gather data on the following patient characteristics: age at the time of surgery in years, sex assigned at birth, race, body mass index (BMI), American Society of Anesthesiologists (ASA) score, and smoking status. Patient records were also reviewed to identify patients' medical history and comorbidities, such as history of hypertension, prior ipsilateral elbow fracture, prior ipsilateral elbow dislocation, or any prior elbow surgery. Additionally, two distinct variables were tracked (1) a prior diagnosis of inflammatory arthritis (eg, psoriatic arthritis or rheumatoid arthritis) and (2) inflammatory arthritis as a primary diagnosis associated with the index surgery. These categories were intentionally tracked as separate variables to distinguish patients with a systemic autoimmune diagnosis from those undergoing contracture release specifically for inflammatory arthritis of the elbow. For example, a patient may have rheumatoid arthritis but undergo surgery due to degenerative changes resulting from post-traumatic arthritis. This approach allowed for more accurate classification of both comorbidities and surgical indications within the cohort. Additional primary diagnoses included osteoarthritis and post-traumatic soft tissue contracture. Lastly, the following patient factors were collected in the immediate preoperative period (<3 weeks prior to surgery): preoperative range of motion, dominant arm procedure, night pain, and presence of ulnar neuritis symptoms. Time between surgery and postoperative PROMIS survey completion (in months) was also noted. Preoperative and postoperative range of motion was measured during an office visit using a goniometer, and neurological function was evaluated through clinical assessment. Any complications or unplanned reoperations during the postoperative period were also noted.

### Patient-reported outcome measures

All subjects completed preoperative and postoperative P-UE, P-Interference, and P-Intensity surveys. PROMIS utilizes a T-score metric, normalized across a scale of 0 to 100, where the reference population mean is 50 with a standard deviation (SD) of 10.^29^ P-UE specifically evaluates activities and motions requiring the use of the upper extremity, with higher scores correlating to increased function.^31^ P-Interference evaluates limitations of motion secondary to pain, while P-Intensity assesses the patient's level of subjective pain.^3^^,^^24^^,^^28^ Higher scores on P-Intensity and P-Interference, respectively, correlate with higher levels of pain and movement limitations secondary to pain.

Patients were divided into 3 quantiles (tertiles) by ordering their postoperative PROMIS scores from highest to lowest, dividing patients into even groups. Poor outcomes were defined as having a P-UE score in the lowest tertile and P-Intensity and/or P-Interference score in the highest tertile. It is important to note that, for these three PROMIS scores, a score of 50 represents the mean for the general population in the United States with a SD of 10.^6^ For example, a person with a P-UE score of 30 is two SDs below the US national mean, suggesting poor function compared to the standard. This standardization is based on calibration testing conducted on a large representative sample of the general population.^6^ Descriptive statistics, including demographic data and mean PROMIS scores within each quantile were calculated to summarize data distribution.

### Statistical analysis

All patient demographics data are represented as means ± SD, while patient history was presented as percentages of the total patient cohort. Primary outcomes were postoperative PROMIS scores. Multivariate logistic regression analyses were performed to evaluate the association between preoperative patient factors and poor postoperative outcomes (ie, bottom tertile of P-UE scores and top tertile for P-Interference and P-Intensity scores). Each regression model was constructed to evaluate 1 preoperative predictor of interest while adjusting for age, sex, and preoperative scores. When female sex was assessed as a predictor of interest, only age and preoperative scores were adjusted for, and vice versa for age. From each model, odds ratios (ORs) with corresponding 95% confidence intervals (CIs) and *P* values were extracted. *P* values were then adjusted for multiple comparisons using the Bonferroni correction.^1^ Preoperative factors with a *P* value of <.05 were considered statistically significant. Specific preoperative factors evaluated include preoperative extension, flexion, supination, and pronation; female sex, age at surgery, BMI, smoking status, ASA class 3, White race, Black race, history of inflammatory arthritis, night pain, a history of prior ipsilateral elbow fracture, history of a prior elbow surgery, ulnar nerve symptoms at the time of surgery, dominant arm surgery, and primary diagnosis of osteoarthritis, inflammatory arthritis, or post-traumatic soft tissue contracture.

In addition, as part of a subgroup analysis, we also evaluated the rate of neurological complications in patients with restricted preoperative flexion. Patients with a preoperative flexion <100° who did not subsequently undergo a PUND were identified, and their postoperative follow-up records were reviewed for any delayed-onset ulnar neuritis or neuropathy symptoms. All statistical analyses were conducted with RStudio version 4.0.3 (Posit, Boston, MA, USA).

## Results

### Patient demographics and outcomes

There were 133 patients identified using the aforementioned Current Procedural Terminology codes: 68 patients were excluded due to having less than 6 months of postoperative PROMIS data or lacking PROMIS data entirely, leaving a final cohort of 65 patients included in the study. PROMIS scores were collected at an average of 51.6 months (SD: 33.7) postoperatively. The mean age at surgery was 51.2 years (SD: 12.1), 28% of patients were female. The average BMI was 28.9 kg/m^2^ (SD: 4.1) (Table I).

**Table I:** Baseline patient demographics and operative details.

Variable	Number (n = 65) Count (%) or mean (range)
Age (yr)	52.2 (19-77)
Sex assigned at birth	
Female	18 (27.7)
Male	47 (72.3)
BMI (kg/m^2^)	28.9 (20.6-37.6)
Race	
White	49 (75.4)
Black or African American	10 (15.4)
Other	6 (9.2)
Smoker	24 (36.9)
ASA	
1	16 (24.6)
2	43 (66.2)
3	6 (9.2)
Comorbidities/history	
Diabetes	3 (4.6)
Hypertension	10 (15.4)
Inflammatory arthritis∗	16 (24.6)
Malignancy affecting the elbow	4 (6.2)
Osteochondritis dissecans	2 (3.1)
Elbow fracture	19 (29.2)
Elbow dislocation	3 (4.6)
Prior elbow surgery	20 (30.8)
Preoperative symptoms	
Night pain	38 (58.5)
Ulnar nerve symptoms at time of surgery	13 (20.0)
Primary diagnosis	
Osteoarthritis	54 (83.1)
Inflammatory arthritis∗	6 (9.2)
Post-traumatic soft tissue contracture	5 (7.7)
Surgery on dominant arm	44 (67.7)
Prophylactic ulnar nerve release performed	7 (10.8)
Time between surgery and PROMIS survey (mo)	51.6 (7-131)

*BMI*, body mass index; *ASA*, American Society of Anesthesiologists; *PROMIS*, Patient-Reported Outcomes Measurement Information System.

∗ Inflammatory arthritis was delineated into two distinct categories: 1) prior diagnoses of a systemic disease and 2) primary diagnosis for surgery. These categories were intentionally separated to distinguish patients with a systemic autoimmune diagnosis from those undergoing contracture release specifically for inflammatory arthritis of the elbow. For example, a patient may carry a diagnosis of rheumatoid arthritis but undergo surgery due to degenerative changes due to posttraumatic arthritis.

Of the patients included in the final analysis, 31% had a history of prior ipsilateral elbow surgery, 29% had a history of an elbow fracture, and 5% had a history of an elbow dislocation. Fifty-four patients (83%) had elbow osteoarthritis as a surgical diagnosis, while 6 patients (9%) had inflammatory arthritis as a surgical diagnosis. Remaining patient characteristics are presented in Table I.

Details on preoperative and postoperative range of motion and PROMIS scores are listed in Table II. Flexion improved from a mean of 122° (range: 80-145) preoperatively to 127° (range: 90-150) postoperatively (*P* = .02), corresponding to a mean improvement of 4.9%. Extension improved from 20° (range: 0-50) to 14° (range: −5 to 45) (*P* < .001), representing a mean improvement of 31.2%. The flexion–extension arc increased from 103° (range: 30-140) to 113° (range: 45-145) (*P* < .001), corresponding to a mean improvement of 16.5%. Supination changed from 79° (range: 10-90) to 81° (range: 40-90), representing a mean improvement of 13.0%, while pronation remained largely unchanged from 85° (range: 50-90) to 85° (range: 50-90), with a mean improvement of 0%.

**Table II:** Preoperative vs. postoperative range of motion and outcome scores.

Variable	Preoperative Mean ± SD	Postoperative Mean ± SD	Change (pre-post) Mean ± SD	*P* value∗
Extension (°)	19.9 ± 13.7	13.9 ± 11.7	−6.0 ± 12.9	**<.001**
Flexion (°)	122.3 ± 14.2	126.9 ± 11.6	4.6 ± 14.8	**.02**
Flexion-extension arc (°)	102.5 ± 22.6	113.1 ± 20.2	10.6 ± 24.4	**<.001**
Supination (°)	79.3 ± 13.0	81.2 ± 8.5	1.9 ± 12.9	.23
Pronation (°)	85.3 ± 7.7	85.0 ± 7.2	−0.3 ± 7.0	.73
P-Intensity Score	54.0 ± 7.5	49.8 ± 6.6	−3.8 ± 9.8	**.01**
P-Interference Score	62.9 ± 7.4	60.2 ± 7.3	−2.2 ± 8.1	.05
P-UE Score	33.5 ± 11.8	38.6 ± 8.4	5.0 ± 13.2	**.01**

*P-UE*, PROMIS, Upper Extremity Function score; *P-Interference*, PROMIS Pain Interference score; *P-Intensity*, PROMIS Pain Intensity score; *SD*, standard deviation; *PROMIS*, Patient Reported Outcome Measurement Information System.

∗ *P* < .05 bolded to indicate statistical significance. Paired *t*-tests were used for significance testing between preoperative and postoperative values.

P-UE scores increased from a mean of 33.5 ± 11.8 preoperatively to 38.6 ± 8.4 postoperatively (*P* = .01). P-Interference scores decreased from 62.9 ± 7.4 to 60.2 ± 7.3 (*P* = .05). P-Intensity scores decreased from 54.0 ± 7.5 to 49.8 ± 6.6 (*P* = .01) (Table II).

### Preoperative factors associated with the poorest outcome scores

Tertile distribution of P-UE, P-Interference, and P-intensity scores are summarized in Table III. After adjusting for multiple comparisons, female sex (OR: 10.7; 95% CI: 2.7-52.4) remained significantly associated with higher odds of having a bottom tertile P-UE score (*P* = .02). History of inflammatory arthritis was associated with increased odds of a bottom-tertile postoperative P-UE score (OR 4.5, 95% CI: 1.1-20.6), though this association was no longer significant after adjustment for multiple comparisons (Table IV).

**Table III:** Comparison of PROMIS scores between tertiles.

Outcome score	Group	Number of patients	Mean score ± SD	Range	*P* value∗
P-UE	Bottom tertile	22	30.3 ± 4.2	21.6-35.4	**<.001**
	Top 2 tertiles	43	42.9 ± 6.6	36.0-61.0	
P-Interference	Bottom 2 tertiles	44	56.6 ± 5.7	38.7-57.0	**<.001**
	Top tertile	21	67.8 ± 3.4	64.4-76.3	
P-Intensity	Bottom 2 tertiles	44	46.7 ± 5.0	30.7-52.3	**<.001**
	Top tertile	21	56.4 ± 4.5	53.7-72.0	

*P-UE*, PROMIS Upper extremity Function score; *P-Interference*, PROMIS Pain Interference score; *P-Intensity*, PROMIS Pain Intensity score; *SD*, standard deviation; *PROMIS*, Patient-Reported Outcomes Measurement Information System.

∗ *P* < .05 bolded to indicate statistical significance.

**Table IV:** Multivariate logistic regressions evaluating risk factors for the lowest postoperative P-UE tertile; each predictor adjusted for age, sex, and preoperative P-UE score.

Predictor	Odds ratio	95% CI	*P* value∗	Adjusted *P* value†
Age (yr)‡	1.02	0.96-1.08	.54	1.00
BMI (kg/m^2^)	1.14	0.94-1.42	.20	1.00
Female sex§	10.73	2.68-52.42	**.001**	**.02**
Preoperative ROM				
Flexion	1.00	0.96-1.05	.89	1.00
Extension	0.98	0.93-1.03	.35	1.00
Supination	0.94	0.87-1.00	.05	1.00
Pronation	0.95	0.87-1.02	.17	1.00
Smoker	0.66	0.15-2.60	.56	1.00
ASA class 3	1.08	0.12-8.93	.94	1.00
Race				
White	0.87	0.19-4.41	.86	1.00
Black or African American	2.24	0.41-11.74	.34	1.00
Comorbidities/history				
Inflammatory arthritis	4.51	1.08-20.62	**.04**	.80
Elbow fracture	2.75	0.63-12.08	.17	1.00
Prior elbow surgery	1.40	0.31-6.19	.65	1.00
Preoperative symptoms				
Night pain	0.98	0.23-4.01	.98	1.00
Ulnar nerve symptoms at time of surgery	2.21	0.50-9.71	.29	1.00
Primary diagnosis				
Osteoarthritis	0.95	0.16-6.40	.95	1.00
Inflammatory arthritis	5.79	0.43-174.63	.23	1.00
Post-traumatic soft tissue contracture	0.27	0.03-1.39	.15	1.00
Surgery on dominant arm	0.35	0.07-1.48	.16	1.00

*ROM*, range of motion; *ASA*, American Society of Anesthesiologists; *CI*, confidence interval; *BMI*, body mass index; *P-UE*, PROMIS Upper extremity function score; *PROMIS*, Patient Reported Outcome Measurement Information System.

∗ *P* < .05 bolded to indicate statistical significance.

† *P* values for risk factors were adjusted for multiple comparisons using the Bonferroni correction.

‡ Sex and preoperative P-UE scores were controlled for.

§ Age and preoperative P-UE scores were controlled for.

No preoperative risk factors of interest were found to be associated with higher odds of a top tertile P-Interference score (Table V). History of inflammatory arthritis was associated with higher odds of a top-tertile P-Intensity score (OR 6.8; 95% CI: 1.7-32.6), though this association was not significant after adjustment for multiple comparisons (Table VI).

**Table V:** Multivariate logistic regressions evaluating risk factors for the highest postoperative P-Interference tertile; each predictor adjusted for age, sex, and preoperative P-Interference score.

Predictor	Odds ratio	95% CI	*P* value∗	Adjusted *P* value†
Age (yr)‡	0.99	0.94-1.04	.78	1.00
BMI (kg/m^2^)	1.06	0.88-1.28	.54	1.00
Female sex§	1.74	0.46-6.53	.41	1.00
Preoperative ROM				
Flexion	1.03	0.98-1.07	.25	1.00
Extension	0.97	0.92-1.01	.17	1.00
Supination	0.99	0.93-1.05	.70	1.00
Pronation	0.95	0.87-1.03	.24	1.00
Smoker	2.29	0.67-8.19	.19	1.00
ASA class 3	0.61	0.07-4.07	.62	1.00
Race				
White	0.84	0.21-3.54	.80	1.00
Black or African American	0.66	0.11-3.15	.61	1.00
Comorbidities/history				
Inflammatory arthritis	1.61	0.39-6.53	.50	1.00
Elbow fracture	0.79	0.19-3.00	.73	1.00
Prior elbow surgery	1.23	0.33-4.39	.75	1.00
Preoperative symptoms				
Night pain	1.46	0.40-5.48	.57	1.00
Ulnar nerve symptoms at time of surgery	**0.09**	**0.01-0.55**	**.02**	.40
Primary diagnosis				
Osteoarthritis	4.47	0.71-41.73	.14	1.00
Inflammatory arthritis	0.64	0.05-6.67	.71	1.00
Post-traumatic soft tissue contracture	0.46	0.09-1.94	.31	1.00
Surgery on dominant arm	0.65	0.18-2.33	.51	1.00

*ROM*, range of motion; *ASA*, American Society of Anesthesiologists; *CI*, confidence interval; *BMI*, body mass index; *P-Interference,* PROMIS pain interference score; *PROMIS*, Patient Reported Outcome Measurement Information System.

∗ *P* < .05 bolded to indicate statistical significance.

† *P* values for risk factors were adjusted for multiple comparisons using the Bonferroni correction.

‡ Sex and preoperative P-Interference score were controlled for.

§ Age and preoperative P-Interference scores were controlled for.

**Table VI:** Multivariate logistic regressions evaluating predictors of landing in the highest postoperative P-Intensity tertile; each predictor adjusted for age, sex, and preoperative P-Intensity score.

Predictor	Odds ratio	95% CI	*P* value∗	Adjusted *P* value†
Age (yr)‡	0.97	0.92-1.01	.17	1.00
BMI (kg/m^2^)	0.84	0.68-1.01	.08	1.00
Female sex§	2.63	0.70-10.26	.15	1.00
Preoperative ROM				
Flexion	1.02	0.98-1.07	.30	1.00
Extension	0.97	0.93-1.02	.22	1.00
Supination	0.98	0.92-1.04	.44	1.00
Pronation	1.00	0.93-1.08	.95	1.00
Smoker	0.79	0.22-2.67	.71	1.00
ASA class 3	2.72	0.38-19.86	.30	1.00
Race				
White	1.03	0.25-4.65	.97	1.00
Black or African American	0.62	0.08-3.21	.59	1.00
Comorbidities/history				
Inflammatory arthritis	6.80	1.65-32.58	**.01**	.20
Elbow fracture	0.83	0.19-3.22	.80	1.00
Prior elbow surgery	1.01	0.26-3.64	.99	1.00
Preoperative symptoms				
Night pain	0.96	0.26-3.44	.95	1.00
Ulnar nerve symptoms at time of surgery	0.40	0.07-1.80	.27	1.00
Primary diagnosis				
Osteoarthritis	0.80	0.16-4.34	.79	1.00
Inflammatory arthritis	5.96	0.58-142.61	.17	1.00
Post-traumatic soft tissue contracture	0.48	0.09-2.09	.34	1.00
Surgery on dominant arm	1.90	0.52-8.06	.35	1.00

*ROM*, range of motion; *ASA*, American Society of Anesthesiologists; *CI*, confidence interval; *BMI*, body mass index; *P-Interference,* PROMIS pain interference score; *PROMIS*, Patient Reported Outcome Measurement Information System.

∗ *P* < .05 bolded to indicate statistical significance.

† *P* values for risk factors were adjusted for multiple comparisons using the Bonferroni correction.

‡ Sex and preoperative P-Intensity scores were controlled for.

§ Age and preoperative P-Intensity scores were controlled for.

### Complications and reoperations

Three patients (4.6%) required reoperation following the index procedure. Two patients underwent delayed ulnar nerve transposition at 4 months and 6 years postoperatively due to persistent ulnar nerve symptoms. One patient with primary osteoarthritis underwent repeat arthroscopic release for recurrent stiffness at six months postoperatively, followed by conversion to total elbow arthroplasty two years later for ongoing degenerative changes.

Three patients experienced persistent, though improved, ulnar nerve symptoms, which were treated nonoperatively. Three other patients developed new-onset ulnar neuropathy symptoms, all of whom were treated nonoperatively. Two of those three experienced worsening symptoms over 1-2 years, while one reported symptom relief with corticosteroid injections. One additional patient developed a postoperative infection requiring arthroscopic irrigation and débridement.

### Neurological complications in patients with preoperative flexion of <100°

A subgroup of seven patients within the cohort presented with limited preoperative elbow flexion (<100°), with an average preoperative flexion of 95.7°. All seven patients had elbow osteoarthritis as their surgical indication, and none underwent a PUND at the time of surgery. At an average in-person follow-up of 13 months, one patient developed new-onset ulnar nerve symptoms that showed partial improvement but remained persistent and were managed with nighttime splinting and nerve gliding. No other neurological complications were reported within this subgroup (Table VII).

**Table VII:** Characteristics and outcomes of patients with preoperative flexion <100°.

Patient #	Preoperative ROM		Postoperative ROM		Primary diagnosis	PUND performed	Postoperative ulnar symptoms
	Flexion	Extension	Flexion	Extension			
1	100°	40°	100°	40°	OA	No	No
2	90°	10°	120°	10°	OA	No	No
3	100°	40°	120°	30°	OA	No	No
4	100°	5°	135°	0°	OA	No	Yes
5	90°	30°	120°	15°	OA	No	No
6	90°	5°	105°	0°	OA	No	No
7	100°	30°	120°	20°	OA	No	No

*ROM*, range of motion; *PUND*, prophylactic ulnar nerve decompression; *OA*, osteoarthritis.

## Discussion

The principal finding of this study is that a majority of patients who underwent arthroscopic intervention for elbow contractures experienced improvements in pain and function levels, as evidenced by satisfactory PROMIS outcomes and range of motion, accompanied by a low incidence of complications. Notably, female sex was found to be associated with poor postoperative function.

In an editorial commentary entitled “Arthroscopic Release of Severe Elbow Contracture Is Safe and Effective: It Can Be Done, and It Should Be Done,” Sheean^26^ discussed the success of a single surgeon treating elbow contractures arthroscopically, highlighting the importance of further research to validate the efficacy and complications of this procedure. In the original study, in which Beck et al^2^ followed 52 patients for an average follow-up of over 5 years, 8 patients (15.4%) required revision surgery for failure of improvement of range of motion, a rate notably higher than that observed in our study at 4.6% (3 out of 65). Schreiner et al^25^ studied 44 patients with post-traumatic elbow stiffness who underwent arthroscopic arthrolysis and found significant improvement in range of motion with a very low complication rate of 2%. Furthermore, Wu et al^33^ studied 34 patients and found a complication rate of 5.8% with no patients requiring revision surgery. Each of these studies demonstrates the overall success of arthroscopic arthrolysis for the treatment of symptomatic elbow contracture.

Prior studies have investigated risk factors for treatment failure following arthroscopic treatment of elbow contractures. Schreiner et al^25^ found preoperative pain levels to be significantly associated with worse postoperative outcome measurements. Noticewala et al^21^ studied the 30-day postoperative complication profile following elbow arthroscopy in 530 patients and found renal disease, preoperative steroid use, and higher ASA class to be risk factors for adverse events and treatment failure. Importantly, the study included all indications for elbow arthroscopy.^21^

To our knowledge, this is the first study to highlight female sex as a potential risk factor for inferior patient-reported outcomes, specifically P-UE scores, following arthroscopic treatment for elbow contractures. Intravia et al^15^ studied 560 elbow arthroscopies and found female sex to be associated with a significantly higher risk for nerve injury but found no correlation with other patient-reported outcomes following surgery. Furthermore, Tsenkov and Dimitrov's^30^ systematic review, which included over 50 publications on elbow arthroscopy, identified female sex as a risk factor for an increased all-cause complication rate, along with comorbidities such as obesity, diabetes, tobacco and alcohol use, and advanced age (≥65 years). It is important to consider that the P-UE questionnaire assesses various aspects of upper extremity function related to activities of daily living, including tasks such as putting on clothes and brushing one's hair. This particular item may have disproportionately affected female participants, potentially contributing to lower overall scores in this subgroup.

Management of the ulnar nerve during arthroscopic ECR remains a topic of debate, particularly in patients with restricted preoperative flexion. One such consideration is the role of PUND, which is often performed in patients with flexion deficits ≤100°.^27^^,^^32^ Some surgeons advocate for routine decompression in this setting to reduce the risk of postoperative neuropathy, as elbow contractures can lead to adaptive shortening of the ulnar nerve, increasing susceptibility to traction neuritis as motion improves postoperatively.^4^^,^^32^ In our subgroup analysis, seven patients underwent arthroscopic ECR with preoperative flexion <100° without PUND. Only 1 patient developed new-onset postoperative ulnar nerve symptoms. While this suggests that selective decompression may be a reasonable approach in carefully selected patients, the small sample size severely limits the strength of this conclusion, and larger studies are needed to guide management in this population.

This study has several important limitations. First, its retrospective design introduces inherent risks of selection bias and precludes the ability to infer causal relationships, allowing only for the identification of associations. Second, a substantial number of patients were excluded due to incomplete PROMIS follow-up (<6 months), which may reduce statistical power and introduce selection bias. Another limitation is that “inferior outcomes” were defined using tertile stratification based on the cohort's score distribution, as validated minimal clinically important difference, patient acceptable symptom state, and substantial clinical benefit thresholds for PROMIS outcomes following arthroscopic ECR have not yet been established. However, because PROMIS T-scores are standardized to a national reference population (mean 50, SD 10), patients classified in our lowest P-UE tertile represent individuals with objectively poor upper-extremity function (2 SDs below the mean) relative to the general population, not solely relative to this cohort. Additionally, PROMIS outcomes were collected at variable postoperative time points, which may introduce heterogeneity in patient-reported outcomes and limit the consistency of follow-up assessment. Our analysis of inflammatory arthritis as a comorbidity was not stratified by specific diagnoses, limiting the diagnostic granularity of our findings. Although a potential interaction between variables that demonstrated significant associations in our analysis, such as female sex and inflammatory arthritis, is biologically plausible, the limited number of patients with inflammatory arthritis—particularly female patients (6 out of 16)—precluded reliable interaction modeling. Next, although the procedures were performed by fellowship-trained surgeons using a similar arthroscopic approach, subtle variations in surgical technique and postoperative rehabilitation protocols may have existed and represent a potential source of unmeasured confounding.

## Conclusion

Patients with elbow contracture due to various underlying pathologies may experience meaningful improvements in function and range of motion following arthroscopic contracture release, with a low reoperation rate. However, certain preoperative factors, such as female sex, may be associated with worse postoperative functional scores.

## Disclaimers

Funding: No funding was disclosed by the authors.

Conflicts of interest: Mandeep S. Virk is a paid consultant for Exactech, Inc. Young W. Kwon is a paid consultant for DJO Surgical. The other authors, their immediate families, and any research foundations with which they are affiliated have not received any financial payments or other benefits from any commercial entity related to the subject of this article.
